# Frank’s Sign as a Dose-Dependent Marker of White Matter Burden in CADASIL: A Brain MRI Study

**DOI:** 10.3390/jcm14196865

**Published:** 2025-09-28

**Authors:** Sungman Jo, Joon Hyuk Park, Ki Woong Kim

**Affiliations:** 1 Department of Health Science and Technology, Graduate School of Convergence Science and Technology, Seoul National University, Seoul 08826, Republic of Korea; josman89@snu.ac.kr; 2 Department of Psychiatry, Jeju National University School of Medicine, Jeju 63243, Republic of Korea; 3 Department of Neuropsychiatry, Jeju National University Hospital, Jeju 63241, Republic of Korea; 4 Department of Neuropsychiatry, Seoul National University Bundang Hospital, Seongnam 13620, Gyeonggi-do, Republic of Korea; 5 Department of Psychiatry, Seoul National University College of Medicine, Seoul 03080, Republic of Korea; 6 Department of Brain and Cognitive Science, Seoul National University College of Natural Sciences, Seoul 08826, Republic of Korea; 7 Institute of Human Behavioral Medicine, Seoul National University Medical Research Center, Seoul 03080, Republic of Korea

**Keywords:** CADASIL, Frank’s sign, white matter hyperintensities, cerebral small vessel diseases, magnetic resonance imaging

## Abstract

**Background/Objectives:** Frank’s sign, a diagonal earlobe crease, may reflect systemic microvascular dysfunction. We investigated whether Frank’s sign serves as a clinical marker of white matter hyperintensity (WMH) burden in Cerebral Autosomal Dominant Arteriopathy with Subcortical Infarcts and Leukoencephalopathy (CADASIL), a monogenic model of pure cerebral small vessel disease. **Methods:** We analyzed 81 genetically confirmed CADASIL patients (61.8 ± 12.6 years, 40.7% female) and 54 age/sex-matched controls (70.3 ± 6.6 years, 48.1% female). Frank’s sign was detected using deep learning from brain MRI-reconstructed 3D facial surfaces. WMH volumes were automatically quantified and adjusted for confounders using Random Forest regression residuals. We compared Frank’s sign prevalence between groups, assessed within-CADASIL associations, and evaluated dose–response relationships across WMH tertiles. **Results:** Frank’s sign prevalence was significantly higher in CADASIL versus controls (66.7% vs. 42.6%, *p* = 0.020), with strengthened association after multivariate adjustment (OR = 4.214, 95% CI: 1.128–15.733, *p* = 0.032). Within CADASIL, Frank’s sign-positive patients showed 72% greater WMH burden (51.5 ± 27.1 vs. 30.0 ± 26.1 mL, *p* < 0.001) and lower Consortium to Establish a Registry for Alzheimer’s Disease (CERAD) total scores (57.7 ± 19.6 vs. 71.2 ± 22.8, *p* = 0.006), but similar lacunes, microbleeds, and hippocampal volumes. A robust dose–response relationship emerged across WMH tertiles, with Frank’s sign prevalence increasing from 37.0% (lowest) to 74.1% (highest tertile; adjusted OR = 3.571, 95% CI: 1.134–11.253, *p* = 0.030). **Conclusions:** Frank’s sign represents an accessible biomarker of WMH burden in CADASIL, demonstrating disease-specificity and dose–response characteristics independent of vascular risk factors. The automated MRI-based detection method of Frank’s sign enables retrospective analysis of existing neuroimaging databases, transforming a bedside observation into a quantifiable neuroimaging biomarker for genetic small vessel disease stratification.

## 1. Introduction

Frank’s sign (FS), a diagonal earlobe crease extending from the tragus to the outer ear border, has emerged as an unexpected marker of cerebrovascular disease [1,2,3]. While traditionally associated with coronary artery disease, recent evidence suggests its relevance to cerebral small vessel disease (SVD), which accounts for 25% of all strokes and represents the leading cause of vascular cognitive impairment [4]. Despite the critical need for accessible biomarkers to identify and stratify SVD patients, current diagnostic approaches rely heavily on advanced neuroimaging that typically reflects late-stage disease. The potential of easily observable physical signs to indicate underlying cerebral pathology remains largely unexplored.

The pathophysiological basis linking FS to cerebral SVD involves shared vulnerability of end-arterial systems. Both the earlobe and cerebral microvasculature lack collateral circulation, rendering them susceptible to chronic hypoperfusion [5]. Histopathological studies demonstrate myoelastofibrosis in arterial vessels at the base of the earlobe crease, mirroring the arteriolosclerosis characteristic of cerebral SVD [6]. Furthermore, patients with FS exhibit molecular signatures of vascular aging, including shortened telomeres and reduced Klotho levels, biomarkers also implicated in cerebral SVD progression [1,7].

Recent neurological studies have established FS as a potential cerebrovascular biomarker with clinical relevance. Among cognitively impaired patients, FS confers 2.4-fold increased odds of subcortical vascular cognitive impairment (Odds Ratio [OR] = 2.38, 95% confidence interval [CI] 1.56–3.63) [2]. In a prospective cohort of patients with acute stroke, the prevalence of FS was reported to be as high as 78.8% [8]. Another large study focused on its prognostic value, demonstrating that the presence of bilateral, deep earlobe creases independently predicted poor functional outcomes at 90 days (OR = 1.87, 95% CI: 1.13–3.09) [9]. Furthermore, FS prevalence has been shown to differ by stroke subtype, being more common in ischemic than in hemorrhagic stroke [10]. However, these associations have been examined primarily in heterogeneous populations with sporadic cerebrovascular disease, where multiple confounding factors complicate interpretation. Recent advances in deep learning enable automated detection of FS from 3D facial surfaces reconstructed from routine brain MRI without an inter-observer variability [11].

Cerebral Autosomal Dominant Arteriopathy with Subcortical Infarcts and Leukoencephalopathy (CADASIL), caused by NOTCH3 mutations, provides an ideal model to study pure SVD pathophysiology without confounding atherosclerotic factors [12,13,14,15,16]. This monogenic disorder manifests characteristic white matter hyperintensities (WMH) that progressively accumulate, offering a unique opportunity to examine whether FS correlates with genetically determined cerebral SVD burden. Recent evidence suggests that NOTCH3 mutations affect not only cerebral vessels but also systemic arteries [17], raising the possibility that peripheral vascular stigmata might reflect central nervous system pathology.

We hypothesized that if FS truly reflects systemic microvascular dysfunction rather than merely atherosclerotic burden, it should: (1) be more prevalent in CADASIL patients compared to age-matched controls; (2) correlate specifically with WMH volume, the hallmark of CADASIL severity; and (3) demonstrate a dose–response relationship with disease burden independent of traditional vascular risk factors. This study investigates these hypotheses using deep learning-based FS detection and automated WMH quantification in genetically confirmed CADASIL patients, testing whether this simple physical finding can serve as a clinical marker of underlying white matter pathology in hereditary SVD.

## 2. Methods

### 2.1. Subjects

This retrospective cross-sectional study enrolled all 81 eligible patients (age range: 34–86 years) with genetically confirmed CADASIL from Jeju National University Hospital between 2018 and 2023. Thus, the sample size reflects the entire eligible CADASIL cohort during the study period. CADASIL diagnosis was established through NOTCH3 gene sequencing (*n* = 73) or skin biopsy demonstrating granular osmiophilic material (*n* = 8) [18,19].

For the case–control analysis to examine the association between FS and CADASIL, we recruited age- (±3 years) and sex-matched controls from the Korean Longitudinal Study on Cognitive Aging and Dementia (KLOSCAD) [20] and the visitors to the dementia clinic at Seoul National University Bundang Hospital between 2018 and 2024. All control participants were cognitively normal with the Clinical Dementia Rating (CDR) [21] of 0, living independently in the community, and had no previous history of stroke. From our full cohort of 81 CADASIL patients, 54 were included in the case–control analysis based on the availability of suitable age- and sex-matched controls.

The full cohort of 81 patients was used for all intra-CADASIL group analyses to examine the association between FS and the clinical and neuroimaging characteristics of CADASIL.

The study was approved by the Institutional Review Board (IRB) of Jeju National University Hospital and Seoul National University Bundang Hospital (IRB Nos. JEJUNUH 2025-03-009 and B-2005-615-001).

### 2.2. Clinical Assessments

All CADASIL patients and normal controls underwent comprehensive clinical and neuropsychological evaluations. Board-certified geriatric psychiatrists with expertise in dementia research conducted standardized diagnostic assessments including medical history reviews, physical examinations, and neurological evaluations based on the Korean version of the Consortium to Establish a Registry for Alzheimer’s Disease Clinical Assessment Battery (CERAD-K-C) [22]. Cognitive function was assessed by trained neuropsychologists or certified nurses using the Korean version of the CERAD Neuropsychological Assessment Battery (CERAD-K-N) [23]. Global cognitive function was quantified using the CERAD total score (CERAD-TS) [24] and Mini Mental Status Examination (MMSE) [25]. Depressive symptoms were evaluated using the Korean version of the Geriatric Depression Scale Short Form (SGDS) [26]. Vascular risk factors (hypertension and diabetes mellitus) were assessed through structured interviews and confirmed by medical records and laboratory findings. History of stroke was assessed by neuroimaging.

Final diagnoses and CDR [21] were determined through consensus by a panel of geriatric psychiatrists. Dementia was diagnosed according to the Diagnostic and Statistical Manual of Mental Disorders, Fourth Edition (DSM-4) [27].

### 2.3. Brain MRI Acquisition

Brain MRI was performed using a 3.0T Philips Achieva scanner (Philips Medical Systems, Best, The Netherlands) at both Jeju National University Hospital and Seoul National University Bundang Hospital. For participants scanned at Jeju National University Hospital, the imaging protocol included the following sequences: 3D FLAIR (VISTA; TR/TE = 4800/320 ms, TI = 1650 ms, turbo factor = 240, 1 × 1 × 1 mm spatial resolution, 1 × 1 × 0.5 mm reconstructed resolution, SENSE factor = 5), 3D T1-weighted imaging (TFE; TR/TE = 8/4 ms, flip angle = 15°, 1 × 1 × 1 mm spatial resolution, 1 × 1 × 0.5 mm reconstructed resolution, SENSE factor = 2), and susceptibility-weighted imaging (SWI; TR/TE = 15/21 ms, flip angle = 15°, FOV = 210 × 210 mm, matrix = 280 × 280, slice thickness = 2 mm, slab thickness = 150 mm, SENSE factor = 2). For participants recruited at Seoul National University Bundang Hospital, the imaging protocol included the following sequences: 3D T1-weighted imaging (TFE; TR/TE = 8.1/4.6 ms, flip angle = 8°, FOV = 240 × 240 mm, matrix = 480 × 480, slice thickness = 1.0 mm with no gap, voxel size = 0.5 × 0.5 × 1.0 mm^3^), and axial FLAIR (TR/TE = 9900/125 ms, flip angle = 90°, slice thickness = 3.0 mm with no gap, matrix = 256 × 256, voxel size = 0.47 × 0.47 × 3.0 mm^3^).

Estimated intracranial volume (eICV) and hippocampal volume were calculated using FreeSurfer 7.0.0 (Martinos Center for Biomedical Imaging, Charlestown, MA, USA). 3D FLAIR images were co-registered to T1-weighted images using affine transformation in SPM12 (Wellcome Institute of Neurology, University College London, London, UK). WMH segmentation was performed using the lesion prediction algorithm within SPM12′s Lesion Segmentation Toolbox (http://www.statistical-modeling.de/lst.html, assessed on 1 May 2025) [28]. Lacunes were defined as CSF-intensity signal voids ≥2 mm and ≤15 mm in brain parenchyma, consistent with Standards for Reporting Vascular Changes on Neuroimaging (STRIVE) criteria [29], excluding sub-basal ganglia lesions. Cerebral microbleeds (CMBs) were defined as round signal voids ≤10 mm on SWI, excluding symmetrical basal ganglia hypointensities.

### 2.4. Segmentation of Frank’s Sign

**Figure 1:**
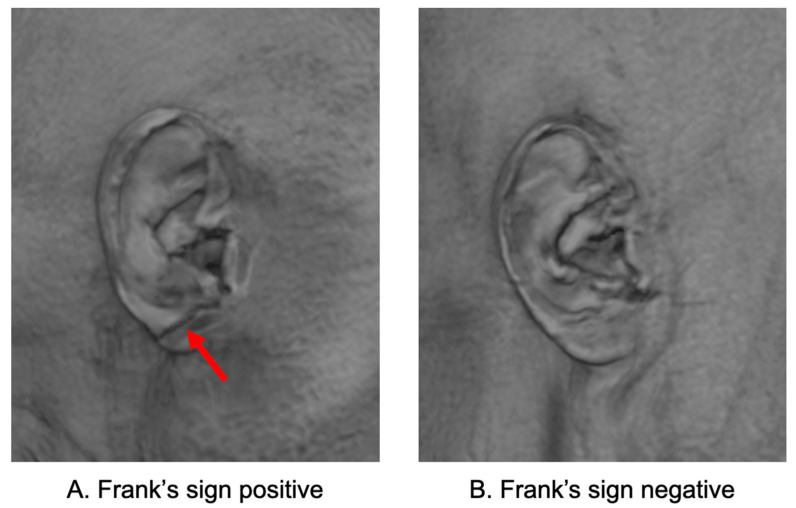
MRI-based visualization of Frank’s sign. (**A**) FS-positive: A diagonal earlobe crease extending from the tragus to the outer ear border (arrow). (**B**) FS-negative: Earlobe without a diagonal crease. Images were derived from 3D surface reconstruction of T1-weighted MRI. FS, Frank’s sign.

FS was automatically identified using a deep learning model developed based on 3D facial surfaces reconstructed from T1-weighted brain MRI scans. The model consists of automated facial surface extraction, ear region localization, and classification using a convolutional neural network trained to detect diagonal earlobe creases. This MRI-based method requires no additional photographs or manual labeling and has been externally validated with high accuracy and clinical applicability [11]. In this study, we directly applied the pretrained model to our dataset without additional training or fine-tuning. FS-positive was defined as the presence of a diagonal earlobe crease detected in at least one ear; therefore, both unilateral and bilateral cases were classified as FS-positive. Representative examples of FS-positive and FS-negative earlobes reconstructed from MRI are shown in Figure 1.

### 2.5. Statistical Analysis

The statistical analysis was designed to comprehensively evaluate the association between FS and WMH burden in CADASIL using a three-stage approach. All analyses were performed using Stata 16.1 (StataCorp LLC, College Station, TX, USA) and Python 3.10, with two-tailed tests and significance set at *p* < 0.05.

To control for potential confounding variables, raw WMH volume was adjusted using a residual approach. We trained a Random Forest regression model to predict WMH volume based on age, sex, years of education, eICV, hypertension, diabetes mellitus, and stroke history. The residuals (observed minus predicted WMH values) were used as covariate-adjusted WMH measures in all subsequent analyses, providing a cleaner assessment of the FS-WMH relationship independent of these confounders. The model’s performance was evaluated using cross-validation to ensure reliable adjustment.

We first compared demographic and clinical characteristics between age- and sex-matched CADASIL patients and cognitively normal controls (*n* = 54 per group). Continuous variables were analyzed using two-way analysis of variance (ANOVA) with diagnosis (CADASIL vs. Normal), FS (present vs. absent), and their interaction as fixed factors. Categorical variables were analyzed using logistic regression models including diagnosis, FS, and their interaction as predictors. This allowed us to examine whether the relationship between FS and clinical features differed between diagnostic groups. The association between CADASIL diagnosis and FS presence was evaluated using logistic regression, with and without covariate adjustment (age, sex, education, eICV, hypertension, diabetes mellitus, and stroke history). OR and 95% CI were computed to quantify the strength of associations.

We then analyzed the full cohort of 81 CADASIL patients to assess whether FS reflects greater WMH burden and other clinical characteristics. Within this cohort, we compared demographic, clinical, cognitive, and neuroimaging characteristics between FS-positive and FS-negative subgroups. Continuous variables were analyzed using independent *t*-tests, while categorical variables were compared using the chi-square test or Fisher’s exact test for small cell counts. Logistic regression was used to estimate OR for FS presence based on WMH volume, both raw and adjusted. To explore the relationships between WMH burden, and clinical characteristics, Pearson correlation coefficients were calculated

To establish a dose–response relationship, we stratified the 81 CADASIL patients into tertiles based on both raw and adjusted WMH volumes. This tertile-based approach was chosen to provide clinically interpretable risk categories while maintaining adequate statistical power in each stratum. FS prevalence was calculated for each tertile. Multivariable logistic regression models were used to compute OR in each tertile, with the first tertile (lowest WMH) as the reference category. The *p*-value for linear trend across tertiles was calculated using logistic regression with tertile as an ordinal variable.

## 3. Results

### 3.1. Association of Frank’s Sign with the Risk of CADASIL

The demographic and clinical characteristics of the 54 CADASIL patients and 54 age- and sex-matched normal controls are presented in Table 1. The two groups were well-matched for age (69.1 ± 7.5 vs. 70.3 ± 6.6 years, *p* = 0.396) and sex distribution (48.1% female in both groups). However, CADASIL patients had significantly lower education levels (7.4 ± 5.1 vs. 13.9 ± 3.0 years, *p* < 0.001). Clinically, a history of stroke was present in 38.9% of patients (vs. 0% in controls), and patients reported more severe depressive symptoms on the SGDS (7.0 ± 5.2 vs. 1.8 ± 1.9, *p* < 0.001). The prevalence of hypertension did not significantly differ between the groups (68.5% vs. 48.1%, *p* = 0.175). On cognitive assessments, CADASIL patients had significantly lower MMSE (21.5 ± 5.2 vs. 28.3 ± 2.0, *p* < 0.001) and CERAD-TS (53.3 ± 18.0 vs. 85.6 ± 12.2, *p* < 0.001), as well as higher CDR-SOB scores (2.5 ± 2.9 vs. 0.0 ± 0.0, *p* < 0.001), with 27.8% of CADASIL patients meeting criteria for dementia.

Neuroimaging parameters revealed striking differences between groups. Raw WMH volume was greater in CADASIL patients (54.2 ± 24.2 vs. 17.2 ± 15.3 mL, *p* < 0.001), with this difference persisting after covariate adjustment (adjusted WMH: 2.8 ± 9.9 vs. −3.1 ± 6.3, *p* < 0.001). Hippocampal volume showed significant atrophy in CADASIL (6.5 ± 0.8 vs. 7.4 ± 0.6 mL, *p* < 0.001), while eICV remained comparable between groups (1555.2 ± 181.4 vs. 1523.3 ± 140.5 mL, *p* = 0.313), confirming that volumetric differences reflected pathological changes rather than constitutional variations.

Finally, FS demonstrated markedly higher prevalence in CADASIL patients compared to controls (66.7% [36/54] vs. 42.6% [23/54], *p* = 0.020). As shown in Table 2, this difference translated to a 2.7-fold increased odds of FS presence in CADASIL (OR = 2.696, 95% CI: 1.234–5.891, *p* = 0.013). This association strengthened to over 4.2-fold after adjusting for demographic and vascular confounders (adjusted OR = 4.214, 95% CI: 1.128–15.733, *p* = 0.032). This persistence after adjustment suggests that FS may reflect intrinsic vascular pathology rather than simply an accumulation of secondary risk factors. In the fully adjusted model, older age (OR = 1.081, *p* = 0.022) and the presence of hypertension (OR = 2.464, *p* = 0.047) were also independently associated with FS.

### 3.2. Association of Frank’s Sign with Disease Severity in CADASIL

Analysis of the full CADASIL cohort (*n* = 81) revealed that FS was present in 48 patients (59.3%, Table 3). Patients with FS were significantly older than those without (65.3 ± 11.3 vs. 56.7 ± 12.8 years, *p* < 0.001), though notably, the age of disease onset did not differ between groups (57.7 ± 12.0 vs. 55.1 ± 10.8 years, *p* = 0.395), suggesting that FS development may be related to disease duration or aging processes rather than early disease manifestation. Patients with FS also had a higher prevalence of hypertension (70.8% vs. 42.4%, *p* = 0.020).

Most notably, patients with FS demonstrated 72% greater WMH volume compared to those without (51.5 ± 27.1 vs. 30.0 ± 26.1 mL, *p* < 0.001). This association remained significant after adjustment for demographic and vascular risk factors (adjusted WMH: 1.6 ± 8.1 vs. −2.2 ± 8.7, *p* = 0.045). In contrast, FS showed no significant associations with other neuroimaging markers: lacune count (5.0 ± 5.8 vs. 3.8 ± 4.2, *p* = 0.395), cerebral microbleeds (11.9 ± 20.0 vs. 5.0 ± 12.3, *p* = 0.128), or hippocampal volume (7.8 ± 1.0 vs. 7.9 ± 1.0 mL, *p* = 0.475).

Patients with FS showed severe depressive symptoms, as measured by the SGDS (6.7 ± 4.8 vs. 4.3 ± 4.8, *p* = 0.030), and lower global cognitive function, as measured by the CERAD-TS (57.7 ± 19.6 vs. 71.2 ± 22.8, *p* = 0.006). The SGDS score and CERAD-TS were correlated with WMH volume (Table 4).

### 3.3. Dose–Response Relationship

A clear dose–response relationship emerged between WMH burden and FS prevalence in the full CADASIL cohort (*n* = 81), with this association strengthening after adjustment for confounding variables (Table 5).

**Figure 2:**
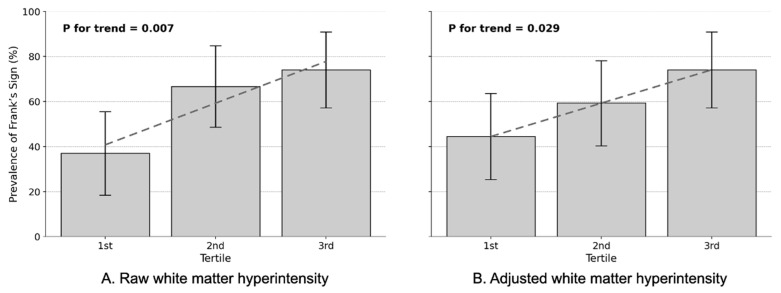
Dose–response relationship between Frank’s sign prevalence and WMH burden in CADASIL patients. Error bars represent standard errors, and gray dashed lines indicate fitted linear trends. Adjusted values represent the residuals from a Random Forest regression model, in which the raw WMH volume was predicted using age, sex, education, estimated intracranial volume, hypertension, diabetes mellitus, and stroke history as covariates.

In the unadjusted analysis, FS prevalence increased progressively across WMH tertiles, from 37.0% in the 1st tertile to 74.1% in the 3rd tertile (*p* for trend = 0.007, Figure 2A). This translated to markedly elevated odds ratios: patients in the highest WMH tertile demonstrated 4.9-fold increased odds of having FS compared to the lowest tertile (OR = 4.857, 95% CI: 1.519–15.530, *p* = 0.008), while the 2nd tertile showed 3.4-fold increases (OR = 3.400, 95% CI: 1.111–10.402, *p* = 0.032). After adjusting for eICV and demographic and vascular confounders, the dose–response pattern persisted with more refined estimates. FS prevalence increased from 37.0% in the 1st tertile to 74.1% in the 3rd tertile across adjusted WMH volume tertiles (*p* for trend = 0.029, Figure 2B). The highest tertile maintained significantly elevated odds (adjusted OR = 3.571, 95% CI: 1.134–11.253, *p* = 0.030), while the 2nd tertile showed a non-significant trend (adjusted OR = 1.818, 95% CI: 0.618–5.352, *p* = 0.278).

Notably, the adjustment revealed the true nature of the FS-WMH relationship. While raw WMH volumes showed strong OR potentially inflated by age and vascular risk factors, the adjusted analysis demonstrated that FS remains an independent marker of white matter pathology. This graded association persisting after controlling for conventional risk factors suggests that FS reflects intrinsic vascular pathology in CADASIL rather than simply serving as a proxy for age or comorbidities.

## 4. Discussion

This study provides the first evidence that FS serves as an independent clinical marker of WMH burden in CADASIL, a monogenic model of pure cerebral SVD. The robust dose–response relationship between FS prevalence and WMH volume tertiles, with prevalence increasing from 37.0% in the lowest tertile to 74.1% in the highest tertile (adjusted OR = 3.571, 95% CI: 1.134–11.253, *p* = 0.030), reveals a previously unrecognized connection between peripheral vascular stigmata and genetically determined cerebral arteriopathy. This finding challenges conventional understanding of FS as merely an atherosclerotic marker and establishes its relevance in hereditary SVD pathophysiology.

### 4.1. Pathophysiological Convergence: From NOTCH3 to Earlobe Creases

The association between FS and WMH burden in CADASIL illuminates shared pathophysiological mechanisms between systemic and cerebral microvasculature. Recent evidence demonstrates that NOTCH3 mutations cause protein aggregation affecting not only cerebral vessels but also systemic arteries including skin, muscle, and potentially earlobe vasculature [14,30]. The accumulation of NOTCH3 extracellular domain (Notch3ECD) leads to progressive arteriopathy through endoplasmic reticulum stress, oxidative damage, and vascular smooth muscle cell degeneration [17,31]. These mechanisms parallel the myoelastofibrosis and arterial wall thickening observed histopathologically in FS-positive earlobe tissue [6].

The specificity of FS to WMH volume, without significant associations with lacunes, cerebral microbleeds, or hippocampal atrophy, suggests that both phenomena reflect diffuse microvascular dysfunction rather than focal ischemic events [12,19]. This aligns with emerging understanding that CADASIL involves heterogeneous white matter pathology, with specific regions showing different water content and microstructural characteristics on advanced 7-Tesla MRI [32]. The parallel vulnerability of end-arterial territories such as the deep white matter and the earlobe further reinforces their shared susceptibility to chronic hypoperfusion [5].

### 4.2. Molecular Mechanisms Linking Peripheral and Central Vascular Pathology

Our findings gain mechanistic support from molecular studies demonstrating accelerated vascular aging in FS. Individuals with FS exhibit shortened telomeres [1] and reduced Klotho levels [7], which have also been associated with the presence, burden, and progression of cerebral SVD [33].

In CADASIL, pericyte dysfunction emerges as a critical pathogenic mechanism [13], potentially explaining the systemic microvascular changes manifesting as FS. The shared vulnerability of pericytes in both cerebral and peripheral microvasculature provides a unifying hypothesis for the observed association. Additionally, observations of shared neurovascular injury processes, such as Wallerian-like degeneration and altered water exchange dynamics in both peripheral tissues and cerebral white matter, further strengthen this proposed link [34].

### 4.3. Clinical Implications: Dose–Response Relationship and Risk Stratification

The dose–response relationship between FS and WMH burden has immediate clinical relevance for CADASIL management. Our tertile analysis revealed a stepwise increase in FS prevalence: 37.0% in the lowest WMH tertile, 66.7% in the middle tertile (OR = 3.400, 95% CI: 1.111–10.402, *p* = 0.032), and 74.1% in the highest tertile (OR = 4.857, 95% CI: 1.519–15.530, *p* = 0.008). After adjustment for confounders, the highest tertile maintained significantly elevated odds (adjusted OR = 3.571, *p* = 0.030), while the middle tertile showed a non-significant trend (adjusted OR = 1.818, *p* = 0.278), suggesting a threshold effect for FS manifestation. Given that WMH volume strongly predicts cognitive decline, stroke risk, and disability progression in CADASIL [19], the doubling of FS prevalence from the lowest to highest WMH tertile provides clinically meaningful stratification. In resource-limited settings or when neuroimaging access is restricted, FS assessment could serve as an initial screening tool to identify patients requiring more intensive monitoring.

### 4.4. Disease Specificity: A General WMH Marker or a Specific Sign of CADASIL

The higher prevalence of FS in CADASIL compared to controls (66.7% vs. 42.6%) is a key finding. The prevalence in our control group (42.6%) is substantially higher than the 14.7% reported in healthy young adults and the 20.3% found in a general cohort of adults being assessed for cardiovascular risk [35,36]. This difference is consistent with the well-established increase in Frank’s sign prevalence with advancing age, suggesting our control group’s prevalence is plausible for an elderly cohort.

This finding, however, leads to a critical question: does FS represent a specific marker of CADASIL pathophysiology, or does it more broadly reflect high WMH burden regardless of etiology? Recent evidence from various clinical populations indeed suggests that FS may serve as a general marker of cerebral SVD. For instance, in a large cohort of cognitively impaired patients, the presence of FS was strongly associated with a higher burden of white matter hyperintensities (OR = 7.29, 95% CI = 3.63 = 14.63) [2]. While another case–control study in ischemic stroke patients found a significant association in unadjusted analyses, this link was no longer statistically significant after adjusting for vascular risk factors [37].

Overall, our findings point toward several features in CADASIL that could suggest disease-specific mechanisms. First, the magnitude and robustness of the association differ substantially. While the link in sporadic stroke did not withstand adjustment for risk factors [37], in our CADASIL cohort the association strengthened to a markedly high OR after comprehensive adjustment (adjusted OR = 4.214). Second, we observed the dose–response relationship in which FS prevalence doubled from the lowest to the highest WMH tertile, a pattern that has not been reported in sporadic SVD. Third, the selectivity for WMH without associations with lacunes or CMBs in our cohort contrasts with sporadic disease, where FS often correlates with multiple SVD markers. However, not all results aligned with this interpretation. Importantly, the non-significant interaction between diagnosis and FS for WMH volume (*p* = 0.189; Table 1) suggests that the FS-WMH relationship may operate similarly across diagnostic groups. This supports the alternative interpretation that FS reflects WMH burden itself, rather than being uniquely specific to CADASIL pathophysiology.

The pathophysiological basis for this enhanced association in CADASIL likely reflects the systemic nature of NOTCH3 arteriopathy [12]. While sporadic WMH arise from heterogeneous mechanisms, CADASIL involves uniform NOTCH3 protein accumulation in both cerebral and peripheral vessels. This relates to another key finding: the similar age of disease onset between FS-positive and negative CADASIL patients (57.7 ± 12.0 vs. 55.1 ± 10.8 years, *p* = 0.395). If FS marked a particularly aggressive subtype of the disease, an earlier onset in the FS-positive group might be expected. The absence of such a difference supports the interpretation that FS is not a marker of a specific disease subtype, but rather a manifestation of the duration and progression of the core, uniform genetic arteriopathy itself. This temporal link to the disease process strengthens its specificity to the underlying NOTCH3 pathology over general vascular risk.

Taking All the above findings into account, our findings thus suggest that while FS may broadly indicate white matter pathology, its manifestation in CADASIL reflects specific NOTCH3-mediated mechanisms that amplify the typical WMH-FS relationship. Future studies comparing CADASIL patients to a control group with a similarly high burden of sporadic WMH would be invaluable to further confirm this disease-specific nature.

### 4.5. Neuropsychiatric Correlates and Frontal-Subcortical Disruption

The neuropsychological profile associated with FS reveals important insights into the cognitive and affective consequences of white matter pathology in CADASIL. Most notably, patients with FS demonstrated significantly lower CERAD-TS (57.7 ± 19.6 vs. 71.2 ± 22.8, *p* = 0.006), indicating impairment across multiple cognitive domains including memory, language, and executive function. This comprehensive cognitive assessment, more sensitive than global screening measures, suggests that FS identifies patients with subtle but widespread cognitive dysfunction that may not be captured by brief screening tools like the MMSE (22.6 ± 5.5 vs. 24.3 ± 4.7, *p* = 0.153).

The CERAD-TS finding is particularly meaningful as it reflects the cumulative burden of cognitive impairment across multiple domains affected by white matter lesions. The 13.5-point difference between FS groups represents a clinically significant effect size, suggesting that FS may serve as a marker for patients requiring comprehensive neuropsychological evaluation. This aligns with the known pattern of cognitive impairment in CADASIL, where executive dysfunction, processing speed deficits, and attention problems predominate over memory impairment alone.

Additionally, the association between FS and depressive symptoms (SGDS scores 6.7 ± 4.8 vs. 4.3 ± 4.8, *p* = 0.030) supports the concept of vascular depression in CADASIL [31]. The disruption of frontal-subcortical circuits by white matter lesions provides a neuroanatomical substrate for both cognitive and affective symptoms. The parallel findings of lower CERAD-TS and higher depression scores in FS-positive patients suggest that this peripheral vascular marker reflects clinically relevant central nervous system pathology affecting both cognitive and emotional regulation networks.

### 4.6. Methodological Innovations and Future Applications

Our use of deep learning-based FS detection from MRI data represents a paradigm shift in clinical phenotyping, eliminating the need for separate ear examinations or photographic documentation [11]. This innovation is particularly significant as it allows retrospective analysis of existing neuroimaging databases without requiring additional clinical assessments. By extracting FS information directly from routine brain MRI scans, we can leverage the vast repositories of neuroimaging data collected for primary neurological evaluation, enabling large-scale epidemiological studies that would be impractical with traditional bedside examination or photography-based methods.

The ability to assess FS solely from brain imaging offers several critical advantages: it eliminates inter-observer variability inherent in subjective clinical assessment, enables standardized evaluation across multiple centers without additional training, and allows for longitudinal tracking of FS changes using existing serial MRI data. This approach transforms FS from a clinical observation requiring dedicated assessment into an automatically extractable neuroimaging biomarker. The successful application in CADASIL demonstrates feasibility for extending this methodology to other genetic SVDs, including Cerebral autosomal recessive arteriopathy with subcortical infarcts and leukoencephalopathy (CARASIL), COL4A1/A2-related disorders, and Fabry disease, where routine MRI monitoring is already standard practice.

Furthermore, the tertile-based analysis strategy we employed provides a clinically interpretable framework for risk stratification. The clear separation between tertiles, with FS prevalence nearly doubling from lowest to highest WMH burden, combined with the automated MRI-based detection method, suggests a practical pathway for integrating this biomarker into routine neuroradiological reporting without additional clinical burden.

### 4.7. Therapeutic Implications and Biomarker Development

The identification of FS as a marker of WMH burden in CADASIL has implications for emerging therapeutic strategies. Recent advances identifying NOTCH3-lowering approaches as promising treatments will require accessible biomarkers for patient selection and treatment monitoring. The dose–response relationship we observed, with FS prevalence tracking WMH tertiles, suggests potential utility as a stratification tool in clinical trials. Furthermore, the systemic nature of vascular changes indicated by FS supports therapeutic approaches targeting both cerebral and peripheral vasculature. The association with molecular aging markers (telomeres, Klotho) also suggests potential therapeutic targets for modulating vascular aging processes in genetic SVD. The 72% greater WMH burden in FS-positive patients provides a clinically meaningful effect size for power calculations in future intervention studies.

### 4.8. Limitations

Several limitations of this study warrant careful consideration. First, the retrospective cross-sectional design precludes causal inference regarding the temporal sequence between FS development and WMH accumulation. Longitudinal studies tracking FS appearance relative to WMH progression are needed to establish whether this peripheral marker precedes, parallels, or follows central nervous system pathology. The absence of data on FS duration or evolution over time further limits our understanding of its natural history in CADASIL.

Second, our sample size, while adequate for primary analyses, may have been underpowered to detect associations with less prevalent neuroimaging markers such as cerebral microbleeds or specific lacune patterns. The non-significant trend toward higher microbleed prevalence in FS-positive patients (11.9 ± 20.0 vs. 5.0 ± 12.3, *p* = 0.128) suggests that larger cohorts might reveal additional associations. Furthermore, we did not assess other peripheral vascular markers such as retinal changes or skin biopsy findings that could provide complementary information about systemic vascular involvement in CADASIL.

Third, the deep learning model for FS detection, while validated in Korean populations, requires external validation in other ethnic groups where earlobe morphology and crease patterns may differ. The model’s performance in detecting subtle or early FS changes remains unknown, potentially leading to misclassification of borderline cases. Additionally, the MRI-based detection method cannot distinguish between congenital earlobe variations and acquired vascular-related creases, though the strong association with WMH burden suggests that most detected cases represent pathological changes.

Fourth, our control group consisted of cognitively normal individuals, which may not fully represent the spectrum of sporadic SVD. Inclusion of patients with varying degrees of sporadic SVD would provide more nuanced insights into the specificity of FS for genetic versus acquired arteriopathy. Moreover, we did not include other monogenic cerebral arteriopathies such as CARASIL or COL4A1/A2-related disorders, limiting conclusions about FS specificity to CADASIL versus other hereditary SVDs.

Finally, we lack data on potential confounding factors such as body mass index, hyperlipidemia, obesity, smoking history, lifestyle factors, or peripheral vascular disease that might influence FS development independently of cerebral pathology. The absence of histopathological correlation between earlobe tissue and cerebral vessels in our cohort also limits mechanistic conclusions about shared vascular pathology. Moreover, because our study cohort consisted exclusively of Korean patients and the overall sample size was modest, caution is warranted when generalizing these findings to other populations or to sporadic forms of SVD.

Future longitudinal studies should investigate whether FS predicts WMH progression rates or clinical outcomes in CADASIL. The potential reversibility of WMH, recently documented in up to one-third of cases [30,38], raises the intriguing possibility that FS might also show dynamic changes correlating with disease activity. Additionally, investigating FS in presymptomatic NOTCH3 mutation carriers could establish its value for early disease detection before irreversible white matter damage occurs.

### 4.9. Conclusions

In conclusion, FS represents a clinically accessible marker of cerebral white matter burden in CADASIL, reflecting shared microvascular pathophysiology between systemic and cerebral arteriopathy. The robust dose–response relationship across WMH tertiles, disease specificity, and independence from traditional vascular risk factors establish FS as more than an epiphenomenon of aging or atherosclerosis. For clinicians managing CADASIL patients, FS assessment should be incorporated into routine examination as a simple indicator of disease severity that may prompt more intensive monitoring or intervention. These findings extend beyond CADASIL to illuminate fundamental connections between peripheral vascular stigmata and cerebral SVD. The clear tertile-based stratification (37.0% → 66.7% → 74.1% prevalence across WMH tertiles) provides a practical framework for clinical application. As precision medicine approaches cerebrovascular disease advance, easily observed physical signs like FS may prove invaluable for risk stratification, treatment selection, and monitoring response to emerging therapies targeting vascular aging and arteriopathy.

## Figures and Tables

**Table 1:** Characteristics of the CADASIL patients and their age- and sex-matched normal controls.

	CADASIL			Normal			*p* *		
	All (N = 54)	FS+ (N = 36)	FS− (N = 18)	All (N = 54)	FS+ (N = 23)	FS− (N = 31)	Dx	FS	Dx × FS
Female	26 [48.1]	19 [52.8]	7 [38.9]	26 [48.1]	12 [52.2]	14 [45.2]	0.669	0.610	0.726
Age, yr	69.1 [7.5]	70.6 [7.1]	66.3 [7.8]	70.3 [6.6]	72.4 [7.2]	68.7 [5.8]	0.396	**0.005**	0.845
Education, yr	7.4 [5.1]	7.0 [5.8]	8.1 [3.6]	13.9 [3.0]	13.8 [2.7]	14.0 [3.3]	**<0.001**	0.472	0.562
APOE ε4	20 [37.0]	14 [38.9]	6 [33.3]	14 [25.9]	4 [17.4]	10 [32.3]	0.938	0.224	0.242
Stroke	21 [38.9]	13 [36.1]	8 [44.4]	0 [0.0]	0 [0.0]	0 [0.0]	**<0.001**	0.616	**<0.001**
Hypertension	37 [68.5]	27 [75.0]	10 [55.6]	26 [48.1]	15 [65.2]	11 [35.5]	0.175	**0.033**	0.676
DM	7 [13.0]	4 [11.1]	3 [16.7]	11 [20.4]	7 [30.4]	3 [9.7]	0.477	0.063	0.094
SGDS	7.0 [5.2]	7.8 [4.7]	5.5 [6.0]	1.8 [1.9]	1.9 [2.1]	1.7 [1.7]	**<0.001**	0.118	0.185
MMSE	21.5 [5.2]	21.4 [5.6]	21.7 [4.2]	28.3 [2.0]	27.8 [2.6]	28.6 [1.4]	**<0.001**	0.476	0.731
CERAD-TS	53.3 [18.0]	51.4 [17.3]	57.2 [19.2]	85.6 [12.2]	84.4 [14.4]	86.5 [10.5]	**<0.001**	0.210	0.536
CDR-SOB	2.5 [2.9]	2.4 [3.0]	2.6 [2.7]	0.0 [0.0]	0.0 [0.0]	0.0 [0.0]	**<0.001**	0.846	0.838
eICV, mL	1555.2 [181.4]	1564.1 [178.9]	1537.2 [190.0]	1523.3 [140.5]	1519.3 [153.6]	1526.3 [132.4]	0.313	0.780	0.604
HPC, mL	6.5 [0.8]	6.5 [0.8]	6.6 [0.7]	7.4 [0.6]	7.3 [0.7]	7.4 [0.6]	**<0.001**	0.305	0.820
WMH, mL	54.2 [24.2]	60.2 [23.2]	42.2 [22.1]	17.2 [15.3]	21.7 [17.9]	13.9 [12.3]	**<0.001**	**0.001**	0.189
aWMH	2.8 [9.9]	5.0 [9.4]	−1.5 [9.6]	−3.1 [6.3]	−2.1 [7.3]	−3.9 [5.4]	**<0.001**	**0.013**	0.145

CADASIL, Cerebral Autosomal Dominant Arteriopathy with Subcortical Infarcts and Leukoencephalopathy; CERAD-TS, total score 2 of Consortium to Establish a Registry for Alzheimer’s Disease Neuropsychological Assessment Battery; FS, Frank’s sign; DM, diabetes mellitus; SGDS, short form Geriatric Depression Scale; APOE, apolipoprotein E; AOD, age of disease onset; MMSE, Mini-Mental State Examination; CDR-SOB, Sum of Boxes of Clinical Dementia Rating; eICV, estimated intracranial volume; HPC, hippocampus; WMH, white matter hyperintensities; aWMH, residuals from a Random Forest regression model, in which the raw WMH volume was predicted using age, sex, education, estimated intracranial volume, hypertension, diabetes mellitus, and stroke history as covariates. Note. Continuous variables are presented as mean [standard deviation] and categorical variables as number [percentage]. Bold values indicate statistical significance at *p* < 0.05. * Analysis of variance for continuous variables and logistic regression analysis for categorical variables.

**Table 2:** Association between CADASIL and Frank’s sign in the age- and sex-matched CADASIL patients and normal controls.

	Unadjusted			Adjusted		
	OR	95% CI	*p*	OR	95% CI	*p*
CADASIL	2.696	1.234–5.891	**0.013**	4.214	1.128–15.733	**0.032**
Age, yr	-	-	-	1.081	1.011–1.156	**0.022**
Sex	-	-	-	0.691	0.246–1.941	0.483
Education, yr	-	-	-	1.035	0.915–1.172	0.582
Hypertension	-	-	-	2.464	1.013–5.991	**0.047**
DM	-	-	-	1.301	0.391–4.322	0.668
Stroke	-	-	-	0.656	0.171–2.519	0.540
Intercept	0.742	0.433–1.272	0.278	0.001	0.000–0.322	0.018

CADASIL, Cerebral Autosomal Dominant Arteriopathy with Subcortical Infarcts and Leukoencephalopathy; DM, diabetes mellitus; OR, odds ratio; CI, confidence interval. Note. Bold values indicate statistical significance at *p* < 0.05.

**Table 3:** Characteristics of the full CADASIL cohort.

	All (N = 81)	FS+ (N = 48)	FS− (N = 33)	*p* *
Age, yr	61.8 [12.6]	65.3 [11.3]	56.7 [12.8]	**<0.001**
Female	33 [40.7]	19 [39.6]	14 [42.4]	0.980
Education, yr	9.6 [5.5]	8.7 [5.8]	11.0 [4.7]	0.059
AOD, yr	56.8 [11.6]	57.7 [12.0]	55.1 [10.8]	0.395
DOI, yr	7.4 [6.9]	8.5 [7.8]	5.3 [4.1]	0.077
Stroke	34 [42.0]	20 [41.7]	14 [42.4]	1.000
Hypertension	48 [59.3]	34 [70.8]	14 [42.4]	**0.020**
DM	12 [14.8]	7 [14.6]	5 [15.1]	1.000
SGDS	5.7 [4.9]	6.7 [4.8]	4.3 [4.8]	**0.030**
APOE ε4 allele	26 [32.1]	17 [35.4]	9 [27.3]	0.597
MMSE	23.3 [5.2]	22.6 [5.5]	24.3 [4.7]	0.153
CERAD-TS	63.2 [21.9]	57.7 [19.6]	71.2 [22.8]	**0.006**
CDR-SOB	1.7 [2.6]	1.9 [2.7]	1.5 [2.3]	0.464
Dementia	15 [18.5]	9 [18.8]	6 [18.2]	1.000
eICV, mL	1564.4 [220.2]	1599.9 [192.8]	1512.8 [248.9]	0.080
WMH, mL	42.8 [28.8]	51.5 [27.1]	30.0 [26.1]	**<0.001**
aWMH	0.0 [8.6]	1.6 [8.2]	−2.2 [8.7]	**0.045**
Lacunes	4.5 [5.3]	5.0 [5.8]	3.8 [4.2]	0.395
Cerebral microbleeds	9.4 [17.8]	11.9 [20.0]	5.0 [12.3]	0.128
Hippocampus, ml	7.8 [1.0]	7.8 [1.0]	7.9 [1.0]	0.475

CADASIL, Cerebral Autosomal Dominant Arteriopathy with Subcortical Infarcts and Leukoencephalopathy; CERAD-TS, total score 2 of Consortium to Establish a Registry for Alzheimer’s Disease Neuropsychological Assessment Battery; DM, diabetes mellitus; SGDS, short form Geriatric Depression Scale; APOE, apolipoprotein E; AOD, age of disease onset; DOI, duration of illness; MMSE, Mini-Mental State Examination; CDR-SOB, Sum of Boxes of Clinical Dementia Rating; eICV, estimated intracranial volume; WMH, white matter hyperintensities; aWMH, residuals from a Random Forest regression model, in which the raw WMH volume was predicted using age, sex, education, estimated intracranial volume, hypertension, diabetes mellitus, and stroke history as covariates. Note. Continuous variables are presented as mean [standard deviation] and categorical variables as number [percentage]. Bold values indicate statistical significance at *p* < 0.05. * Student *t*-test for continuous variables and chi-square test for categorical variables.

**Table 4:** Correlations between the severity of neuropsychiatric symptoms and the volume of white matter hyperintensity.

	Raw WMH Volume		Adjusted WMH Volume *	
	r	*p*	r	*p*
SGDS	0.420	**<0.001**	0.139	0.217
CERAD-TS	−0.646	**<0.001**	−0.224	**0.045**
MMSE	−0.533	**<0.001**	−0.213	0.057
CDR-SOB	0.465	**<0.001**	0.156	0.165

Note: Values represent Pearson correlation coefficients (r) and *p* value. WMH, white matter hyperintensities; SGDS, Geriatric Depression Scale; CERAD-TS, CERAD total score; MMSE, Mini-Mental State Examination; CDR-SOB, Clinical Dementia Rating Sum of Boxes. Note. Bold values indicate statistical significance at *p* < 0.05. * Adjusted values represent the residuals from a Random Forest regression model, in which the raw WMH volume was predicted using age, sex, education, estimated intracranial volume, hypertension, diabetes mellitus, and stroke history as covariates.

**Table 5:** Dose–response relationship between the white matter hyperintensities volume and Frank’s sign in the full CADASIL cohort.

	N (%)	OR [95% CI]	*p*
Raw WMH volume			
1st tertile	10 (37.0)	Reference	-
2nd tertile	18 (66.7)	3.400 [1.111–10.402]	**0.032**
3rd tertile	20 (74.1)	4.857 [1.519–15.530]	**0.008**
Adjusted WMH volume *			
1st tertile	12 (44.4)	Reference	-
2nd tertile	16 (59.3)	1.818 [0.618–5.352]	0.278
3rd tertile	20 (74.1)	3.571 [1.134–11.253]	**0.030**

CADASIL, Cerebral Autosomal Dominant Arteriopathy with Subcortical Infarcts and Leukoencephalopathy; WMH, white matter hyperintensity; ORs, Odds ratio; CI, confidence interval. Note. Bold values indicate statistical significance at *p* < 0.05. * Adjusted values represent the residuals from a Random Forest regression model, in which the raw WMH volume was predicted using age, sex, education, estimated intracranial volume, hypertension, diabetes mellitus, and stroke history as covariates.

## Data Availability

The data underlying this study are available from the corresponding author upon reasonable request. Data sharing is restricted due to ethical and legal limitations imposed by the institutional Review Boards of Jeju National University Hospital and Seoul National University Bundang Hospital.

## Abbreviations

The following abbreviations are used in this manuscript:

CADASIL	Cerebral Autosomal Dominant Arteriopathy with Subcortical Infarcts and Leukoencephalopathy
WMH	White matter hyperintensity
FS	Frank’s sign
SVD	Small vessel disease
